# Single and multi-omic characterization of a porcine model of ethanol-induced hepatic fibrosis

**DOI:** 10.1080/15592294.2025.2471127

**Published:** 2025-03-04

**Authors:** Mark Hieromnimon, Daniel P. Regan, R. Peter Lokken, Lawrence B. Schook, Ron C. Gaba, Kyle M. Schachtschneider

**Affiliations:** a Department of Radiology, University of Illinois at Chicago, Chicago, IL, USA; b Flint Animal Cancer Center, Colorado State University, Fort Collins, CO, USA; c Department of Radiology and Biomedical Imaging, University of California, San Francisco, CA, USA; d Department of Animal Sciences, University of Illinois at Urbana-Champaign, Urbana, IL, USA; e National Center for Supercomputing Applications, University of Illinois at Urbana-Champaign, Urbana, IL, USA; f Sus Clinicals Inc, Chicago, IL, USA; g Department of Biochemistry and Molecular Genetics, University of Illinois at Chicago, Chicago, IL, USA

**Keywords:** Alcoholic liver disease, fibrosis, epigenetics, pig cancer model, translational research

## Abstract

Cirrhosis is a form of end-stage liver disease characterized by extensive hepatic fibrosis and loss of liver parenchyma. It is most commonly the result of long-term alcohol abuse in the United States. Large animal models of cirrhosis, as well as of one of its common long-term sequelae, HCC, are needed to study novel and emerging therapeutic interventions. In the present study, liver fibrosis was induced in the Oncopig cancer model, a large animal HCC model, via intrahepatic, intra-arterial ethanol infusion. Liver sections from five fibrosis induced and five age-matched controls were harvested for RNA-seq (mRNA and lncRNA), small RNA-seq (miRNA), and reduced representation bisulfite sequencing (RRBS; DNA methylation). Single- and multi-omic analysis was performed to investigate the transcriptomic and epigenomic mechanisms associated with fibrosis deposition in this model. A total of 3,439 genes, 70 miRNAs, 452 lncRNAs, and 7,715 methylation regions were found to be differentially regulated through individual single-omic analysis. Pathway analysis indicated differentially expressed genes were associated with collagen synthesis and turnover, hepatic metabolic functions such as ethanol and lipid metabolism, and proliferative and anti-proliferative pathways including PI3K and BAX/BCL signaling pathways. Multi-omic latent variable analysis demonstrated significant concordance with the single-omic analysis. lncRNA’s associated with *UHRF1BP1L* and *S1PR1* genes were found to reliably discriminate the two arms of the study. These genes were previously implicated in human cancer development and vasculogenesis, respectively. These findings support the validity and translatability of this model as a useful preclinical tool in the study of alcoholic liver disease and its treatment.

## Introduction

Hepatic cirrhosis, a form of end-stage liver disease commonly caused by chronic alcohol use, is a significant risk factor for the development of hepatocellular carcinoma (HCC) [1]. Long-term, heavy ethanol consumption, typically on the order of 30 g/d for one to two decades, is known to cause chronic inflammation of the liver, eventually followed by irreversible fibrotic change [2]. Multiple pathomechanisms are responsible for this process, including those associated with hepatic metabolic derangement and direct toxic insult.

Early alcoholic liver disease characteristically first manifests as alcoholic steatosis and steatohepatitis, both of which are in large part driven by metabolic derangement within the liver [3]. The liver is one of the principal sites of lipid metabolism within the human body, and steatosis and steatohepatitis are believed to occur as a result of imbalances in lipid synthesis and breakdown. The genomic aberrations associated with these imbalances are complex and intertwined. One key mechanism is the upregulation of the sterol regulatory element-binding transcription factor 1c (SREBP-1c). SREBP-1c is overexpressed through the direct action of aldehyde, a metabolite of alcohol, and through indirect mechanisms involving cellular stress and inflammatory pathways including Liver X receptor signaling [4]. Factors leading to activation of SREBP-1c also lead to concurrent downregulation of its negative regulators, including AMPK and upregulation of its positive regulators, including STAT3. AMPK serves as an energy balance sensing molecule which is activated by high cellular AMP to ATP ratios and leads to fatty acid oxidation. Alcohol metabolism shifts the balance of this ratio toward increased ATP, suppressing AMPK. STAT3, a transcription factor which is activated by a myriad of inflammatory signaling molecules which are abundant in alcoholic hepatitis including IL-6 and IL-10 and in turn binds to the promoter of the *SREBF1* gene coding for SREBP-1, leading to its transcription [5,6]. Beta-oxidation and steatosis regression are also inhibited through a mechanism involving PPAR-alpha inhibition mediated by direct activity of aldehyde or cytochrome P450 2E1 mediated oxidative stress, among other mechanisms [7]. Together, these changes form the basis for lipid overproduction and the macrovesicular steatosis, hepatocellular ballooning, and ensuing necrosis, which can be observed microscopically as well at the gross steatosis that is often observed clinically in alcoholic liver disease [8].

Concurrent direct toxic insult from acetaldehyde, a metabolite of ethanol, also plays an important role in the pathogenesis of liver fibrosis, cirrhosis, and HCC. Acetaldehyde generates oxidative stress in part through lipid peroxidation within the hepatocyte and within hepatocyte mitochondria. Ensuing hepatocyte damage causes the release of damage associated molecular patterns (DAMPs), which activate innate cellular immunity, generating inflammation with ensuing stellate cell activation. Stellate cell activation in turn leads to hepatic parenchymal replacement with collagen in the wake of hepatocyte death in the setting of chronic ethanol exposure [7].

The epidemiological and public health implications of this disease process are vast. Liver cirrhosis and HCC cause significant morbidity and mortality in both western society and globally. Primary liver cancer, which is predominantly comprised of HCC, is the seventh most frequently occurring cancer in the world and the second most common cause of cancer death [9]. Despite rapid advances in targeted cancer therapies, cancer genotyping, and molecular characterization, the mortality rate for HCC has not commensurately improved, with 5-y survival rates as low as 10% [10]. Historically, HCC has been difficult to treat with systemic chemotherapy or surgical resection due to the myriad of pathologies that emerge in the setting of impaired liver function and decreased hepatic reserve. For example, the hemodynamic derangement and coagulopathy that occur in end-stage liver disease cause significant risk of perioperative mortality or systemic chemotherapeutic toxicity. Novel treatment modalities, including interventional techniques such as transarterial chemo and radioembolization, radiofrequency or microwave ablation, or ethanol injection are valuable therapeutic tools in the clinical arsenal against HCC. These treatments are categorized as locoregional therapies and can function as either curative or palliative interventions in patients with early-stage disease who may not require more aggressive treatment, or in patients with advanced disease who are not candidates for resection or transplantation. Up to 50–60% of HCC patients will be treated at least in part with a locoregional therapy, and these therapies have been shown to delay tumor progression and extend overall survival in early and intermediate stages HCC. These interventions are also frequently employed as bridge therapies for patients who are transplant candidates [11].

Large animal models of alcoholic liver disease are needed for the study and development of new interventional techniques as the currently available small animal models can be technically difficult for operators due to their size, and they do not as closely recapitulate the human pathophysiology of cirrhosis and HCC as pig and non-human primate models [12]. Porcine models are of particular interest in the study of human cancer due to their similar size, anatomy, physiology, genetics, immunity, and metabolism compared to humans. Pigs have a robust innate and adaptive immune system, with many porcine immune proteins having direct human orthologs. In one study, more than 80% of immune parameters analyzed in pig subjects resembled human parameters, compared to 10% in murine subjects [13]. Pig immunology resembles that of *Homo sapiens* to a significant enough degree that they are thought to be the ideal large animal source for human organ xenotransplantation [14]. Porcine models also excel relative to murine models in the realm of pharmaceutical safety and efficacy testing as drugs can be administered to pigs via the oral, intravenous, intraperitoneal, subdermal, inhalational, and intramuscular routes. Furthermore, the structure and function of the cytochrome P450 superfamily of proteins critical for drug metabolism is highly conserved between pigs and humans [15]. Pragmatically speaking, pigs are also an ideal laboratory animal given that they produce sizeable litters, mature quickly, and are more publicly acceptable as laboratory animals than non-human primates as they are also a food source [16]. However, porcine biomedical models have several disadvantages including high housing costs, space requirements, and challenges related to handling due to their large size. In addition, a general lack of commercially available reagents validated for us in porcine studies, including antibodies and genetic tools, limits their utility for biomedical research.

This study utilized the Oncopig, a transgenic porcine cancer model characterized by Cre recombinase-induced TP53^R167H^ and KRAS^G12D^ mutations [17]. Previous studies have demonstrated the ability to induce tumors in Oncopigs that resemble hallmarks of human oncologic imaging, histology, and gene expression profiles. Although rapid, robust, and consistent tumor induction has been demonstrated in this model through direct injection of an adenoviral vector encoding Cre recombinase (AdCre), tumors typically present as undifferentiated carcinomas and/or sarcomas accompanied by significant immune infiltration and spontaneous tumor regression over the course of several weeks or months [18–20]. In addition to direct injection of AdCre, Oncopig hepatocytes have been transformed into HCC cells [21] and autologously injected to induce intrahepatic HCC tumors [22]. Induction of alcoholic liver fibrosis in the Oncopig via intra-arterial ethanol infusion has also been validated to allow for HCC modeling in a clinical relevant comorbid liver microenvironment [23]. However, an understanding of the molecular mechanisms underlying alcoholic liver disease in the Oncopig is required to evaluate the translational relevance of this model. In the present study, liver sections from fibrosis-induced and age-matched control Oncopigs were harvested at 8-weeks post-fibrosis induction for RNA-seq (mRNA and lncRNA), small RNA-seq (miRNA), and reduced representation bisulfite sequencing (RRBS; DNA methylation). Single-omic and multi-omic analysis of mRNA, lncRNA, miRNA, and DNA methylation was performed to investigate the transcriptomic and epigenomic mechanisms associated with fibrosis deposition in this model.

## Methods

### Fibrosis induction

Animal studies and tissue collection were conducted at The University of Illinois at Urbana-Champaign in accordance with national and international guidelines and approved by The University of Illinois Institutional Animal Care and Use Committee (IACUC protocol numbers 10,189 and 10,163). Complete details on animals, housing, and study design can be found in Gaba et al. [23]. Briefly, a single, 10 Oncopig cohort (10 females, 0 males; Sus Clinicals, Inc., Cincinnati, OH) consisting of five pigs subjected to fibrosis induction and five age-matched controls was utilized. Fibrosis induction was performed by board-certified interventional radiologists. Under general anesthesia, hepatic arterial access was accomplished via a common femoral artery approach with the pig in the supine position, after which, a coaxial 3-Fr microcatheter was advanced into the proper hepatic artery where 0.75 mL/kg of 1:3 v/v emulsified mixture of ethanol and ethiodized oil (Lipiodol; Guerbet, Villepinte France) was used to saturate and embolize the hepatic microcirculation with ethanol over the course of 30 min.

### Specimen acquisition

Euthanasia was performed at 8-weeks post-fibrosis induction, with control pigs sacrificed at age-matched timepoints. Necropsy was performed and Oncopig livers were harvested in their entirety. Representative liver samples were acquired from all 10 pigs and transected, with half stored in 10% neutral buffered formalin for pathological analysis and flash frozen in liquid nitrogen within 10 min for transcriptomic and epigenomic analyses. All the samples were stored at −80°C until processing. As described previously, significantly higher fibrosis and inflammation levels were identified in Oncopig fibrotic compared to healthy liver samples using a porcine adapted METAVIR scheme. Peak fibrosis severity was previously demonstrated to occur at 8 weeks post-induction followed by gradual resolution after cessation of alcohol infusion [23,24].

### Illumina sequencing

DNA and RNA were simultaneously isolated from frozen fibrotic (*n* = 5) and control (*n* = 5) liver samples using the AllPrep DNA/RNA Mini Kit (Qiagen, Valencia, CA, USA) and assessed for quality as previously described [23]. High-quality DNA and RNA samples were provided to the Carver High-Throughput DNA Sequencing and Genotyping Unit (HTS lab, University of Illinois, Urbana, IL, USA) for generation of RRBS, rRNA depleted RNA-seq, and small RNA library preparation and sequencing on an Illumina HiSeq4000. RNA-seq libraries were paired-end sequenced (2 × 100 bp), while small RNA and RRBS libraries were single-end sequenced (1 × 50 bp and 1 × 100 bp, respectively). All analyses described below were performed by comparing results from the control liver (*n* = 5) and fibrotic liver samples (*n* = 5).

### RNA-seq analysis

All RNA-seq analysis was performed utilizing standard RNA-seq analysis pipelines utilized for human samples and other preclinical animal studies. Raw RNA-seq reads (average reads per sample: 77795,393; range: 61918,723–89,495,583) were trimmed and analyzed for quality using Trim Galore 0.4.4 (–stringency 6, –illumina, –paired, –retain_unpaired) and assessed for quality using FastQC 0.11.8’s paired end analysis [25,26]. Two-pass alignment to the Sus scrofa reference genome (Sscrofa11.1) was performed using STAR 2.5.3 (–sjdbOverhang 99) using the ensemble 11.1.90 annotation [27,28]. In order to identify novel long non-coding RNAs (lncRNAs), transcripts were first assembled using Cufflinks 2.2.1 [29]. The assembled transcripts were used to identify novel lncRNAs using FEELnc (–biotype=protein_coding; –numtx = 5000,5000; –mode=”shuffle;”–spethres = 0.97,0.97; –proc = 8; –verbosity = 2) [30]. Following integration of lncRNAs into the 11.1.90 annotation, reads were re-aligned to the Sscrofa11.1 genome using STAR 2.5.3 with the same parameters as above. lncRNA classification was performed using the included FEELnc_classifier module. Final quantification of assembled, aligned transcripts was performed using RSEM 1.3.0 (–sjdbOverhang 99) [31]. Differential gene expression analysis was performed using edgeR 3.32 after importing the RSEM quantifications into R using the tximport library [32,33]. edgeR uses an overdispersed Poisson model and empirical Bayes methods to minimize error that can arise from biological and technical variability among samples when identifying differentially expressed genes between groups (Control vs Fibrotic).

### RRBS analysis

All RRBS analysis was performed utilizing standard RRBS analysis pipelines utilized for human samples and other preclinical animal studies. Raw RRBS reads (average reads per sample: 45988,058; range: 42263,129–51,743,942) were trimmed and deduplicated using Trim Galore 0.4.4 (–paired, –length 0) and Nudup, respectively [34]. Diversity adapters were trimmed using python scripts provided by NuGEN Technologies, Inc. (San Carlos, CA) and aligned to the Sus scrofa reference genome (Sscrofa11.1) using BS-seeker 2.1.2 [35]. Methylation calling was performed using BS-seeker 2.1.2 and single nucleotide polymorphisms (SNPs) were identified using BS-snpr [36]. SNP removal and calculation of sequencing depths and conversion rates was performed using in-house scripts. Identification of differentially methylated regions was performed using DMRFinder 0.3 [37]. DMRFinder uses single-linkage clustering of methylation sites into genomic regions with identification of differences in methylation frequencies between groups (Control vs Fibrotic) being performed using beta-binomial hierarchical modeling and Wald tests to account for biological variation among samples. Determination of region-to-gene proximity and region-gene overlap was performed using BEDtools 2.28.0 closestBED and intersectBED functions, respectively.

### miRNA analysis

Raw small RNA-seq reads (average reads per sample: 10957,156; range: 9,540,305–13,317,665) were used for miRNA quantification utilizing the established capMIRSEQ pipeline [38]. Specifically, using default parameters, the capMIRSEQ pipeline was used to perform identification, quantification, and differential expression analysis of known and novel miRNAs aligned to the Sus scrofa 11.1 assembly [38]. capMIRSEQ employs miRDeep2 for miRNA prediction and quantification, which uses Bayesian statistics to score sequenced transcripts and categorize them as miRNAs [39]. Next, capMIRSEQ utilizes edgeR to identify differentially expressed miRNAs between groups (Control vs Fibrotic).

### Multi-omic analysis

Supervised latent variable analysis of expression (mRNA, lncRNA, and miRNA) and DNA methylation regions (collectively referred to as variables) was performed using the DIABLO method within the mixOmics 6.8.5 R library. The DIABLO method is based on the variable selection for generalized canonical correlation analysis (SGCC) and partial least-squares discriminant analysis to perform multivariate dimension reduction suited towards high-dimensional data sets to identify variables accounting for differences among groups (Control vs Fibrotic) [40,41]. A total of 5,000 genes and 5,000 regions with the largest inter-sample variance with non-zero counts in all samples along with all quantified miRNAs were selected for analysis within DIABLO. Cutoffs for the number of genes and regions included in this analysis were based on recommendations from the software developers as described in the mixOmics vignette. These variables were subjected to single component, leave-one-out (loo) validation, and components were assembled using both the top 5 and top 100 most discriminatory variables between fibrotic and control groups as determined by the mixOmics tune.block.splsda function.

### Pathway analysis

Pathway analysis was performed to identify pathways for differentially expressed and differentially methylated genes using Ingenuity Pathway Analysis software (Qiagen Inc, Venlo, Netherlands). miRNAs were submitted for analysis as miRbase human orthologs. Activation z-scores provided by the IPA pathway analysis were used to determine the degree and direction of differential pathway regulation with z-score >2 suggestive of statistically significant upregulation and z-score < −2 suggestive of statistically significant down-regulation. Gene ratio is defined as the number of genes present in the analyzed sample submitted for pathway analysis to the total number of genes known to compose a pathway of interest as determined by IPA.

## Results

### Differential gene expression associated with fibrosis induction

**Figure 1.**
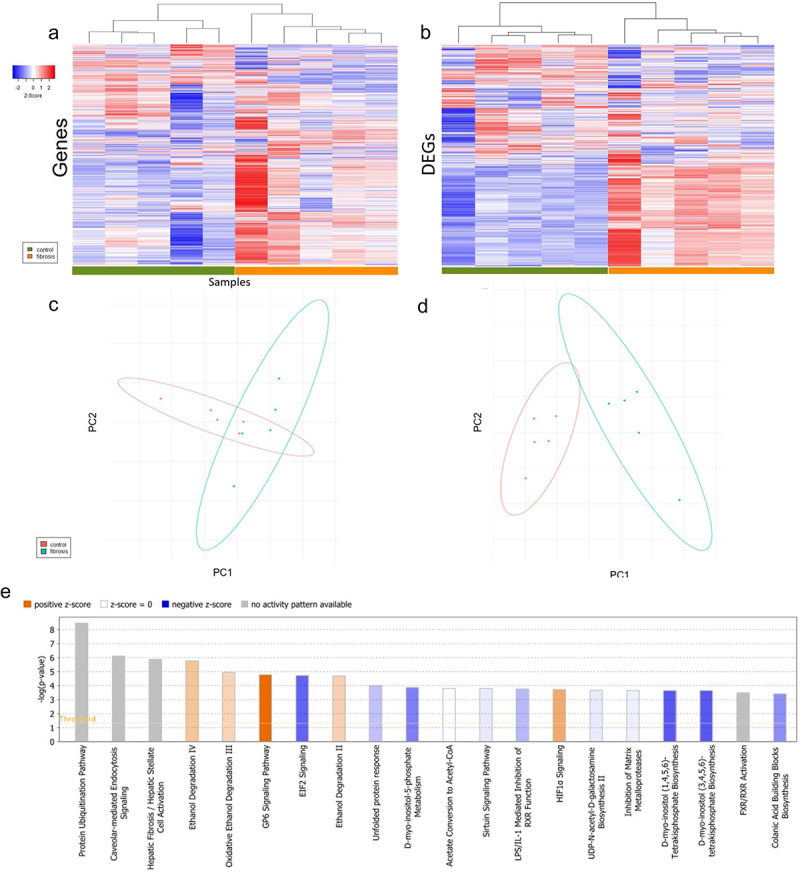
Impact of liver fibrosis induction on gene expression patterns. (a) Hierarchical clustering of subjects based on log fold change of all expressed genes. (b) Hierarchical clustering of subjects based on log change of differentially expressed genes (DEGs). (c) Two-dimensional principle component analysis of subjects based on log fold-change expression of all genes and (d) differentially expressed genes with 95% confidence ellipse. (e) Pathway analysis of differentially expression genes. Positive Z-score indicates predicted over-activation of a given pathway relative to control. Negative Z-score indicates under- activation of a pathway relative to control.

In total, 3,439 differentially expressed genes (DEGs, 2,987 mRNA and 452 lncRNA) were identified between Oncopig fibrotic and control livers. PCA and hierarchical clustering were performed both globally for the 29,226 genes expressed in at least one sample and for DEGs. In both cases, the analyses showed a significant separation of cohorts based on gene expression levels (ANOSIM *R* = 0.308 and 0.776, p-value = 0.023 and 0.007, respectively; Figure 1(a-d)).

DEGs were subjected to pathway analysis for biological characterization of the observed changes in expression. Pathway analysis identified 121 pathways enriched for DEGs (Supplemental Table S1). Of these 121 pathways, hepatic fibrosis, stellate cell activation, and glycoprotein 6 signaling were among the most statistically significant (Figure 1(e)). Differential regulation of the hepatic stellate cell activation and hepatic fibrosis pathways was largely driven by upregulation of 17 collagen isoforms, including *COL12A1, COL15A1, COL16A1*, and *COL1A1*. Differential expression of genes involved in inhibition of matrix metalloproteases was also observed. Most notably, increased expression of *MMP2* and *MMP9* (log2 fold change 2.325 and 6.685, respectively) was observed in fibrotic compared to control Oncopig livers. *TIMP2*, an inhibitor of *MMP2*, was also upregulated in fibrotic compared to control Oncopig livers (log2 fold change 1.916). Enzymatic and oxidative ethanol degradation pathways were also enriched by DEGs, driven by overexpression of alcohol and aldehyde dehydrogenase genes, including *ALDH1A1* and *ADH4*. Multiple isoforms of hydroxysteroid dehydrogenases were also downregulated in Oncopig fibrotic livers, including *HSD17B10, HSD17B7, HSD17B12, HSD17B2, HSD11B1*, and *HSD3B7*.

**Figure 2.**
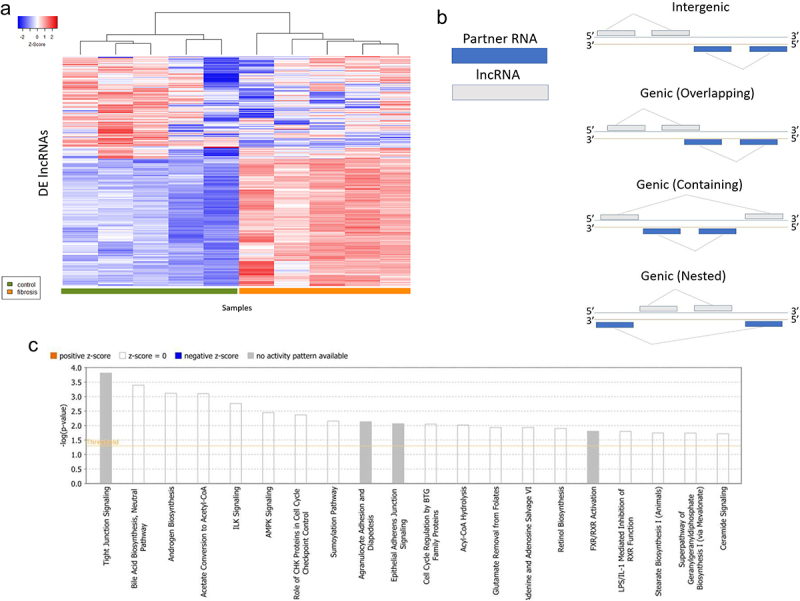
(a) Hierarchical clustering of subjects based on log fold change of differentially expressed long non-coding RNAs. (B) Classification of IncRNA orientation relative to its partner RNA. Adapted from Wucher et al., 2017 (30). (c) Pathway analysis of differentially expressed partner RNA’s to differentially expressed log non-coding RNA’s.

Hierarchical clustering of differentially expressed (DE) lncRNAs showed accurate separation of control and fibrosis groups (Figure 2(a)). Each DE lncRNA was associated with a predicted partner RNA with a total of 368 protein coding partner RNAs identified (mean 2.20 partner transcripts/lncRNA; range 1–26; supplemental Table 2). DE lncRNAs were subclassified as genic or intergenic according to their position with respect to their partner RNA gene and each partner RNA was categorized according to biotype (Figure 2(b)). Of the identified partner RNAs, 83 were DEGs. Pathways enriched for partner DEG’s included bile acid synthesis, androgen synthesis, acetate to acetyl-CoA conversion, and hepatic fibrosis and stellate cell activation (Figure 2(c)). Bile acid synthesis pathway perturbations were largely driven by upregulation of *AKR1C3, AKR1C1*, and *AKR1D1* within the aldo-keto reductase family of proteins in Oncopig fibrotic livers.

### Differential miRNA expression associated with fibrosis induction

**Figure 3.**
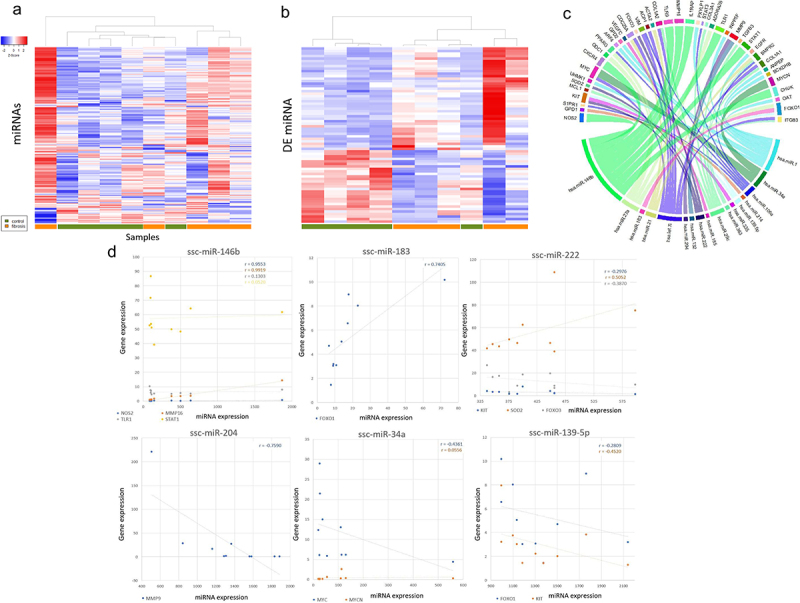
Impact of liver fibrosis induction on miRNA expression patterns. (a) Hierarchical clusting of subjects based on log fold expression of miRNA and (b) log fold expression of differentially expressed miRNA (DEmiRNA). (c) DEGs targeted by DE miRNAs in the Oncopig liver fibrosis model. nChord diagram of differentially expressed micro RNAs and target mRNA expression in the Oncopig liver fibrosis model. Copies per million (CPM) normalized DE miRNA expression versus FPKM normalized DEG expression for DE miRNSs with the greatest magnitude fold-change expression with associated Pearson coefficient.

Of the 326 identified miRNAs, 70 were found to be DE miRNAs. Partial separation of groups was demonstrating when subjecting both all identified miRNAs and DE miRNAs to hierarchical clustering (Figure 3(a,b)). Human orthologs of the DE miRNAs were subjected to target prediction using IPA’s Target Filter software. Following filtering of targets to experimentally observed relationships within hepatic cells, 31 miRNAs targeting 211 mRNAs were identified (mean 9.81 mRNA targets/miRNA; range 1–38; supplemental table S3). Of the 211 mRNAs, 42 were DEGs, including several collagen isoforms, *MMP9*, pro- and anti-proliferative genes, and genes associated with vasculogenesis such as *VEGFC* and *S1PR1* (Figure 3(c)). Pearson’s correlations on the three DE miRNAs with the highest average log fold changes (positive and negative) and their target DEGs demonstrated strong correlations (Figure 3(d)). The most commonly targeted transcripts included *PTEN* (9), COL isoforms (6), *CDK6* (6), *BCL2* (4), and *BCL2L* isoforms (4). More specifically, ssc-let-7i and ssc-miR-29c were upregulated in Oncopig fibrotic livers and found to target *COL1A1, COL1A2, and COL3A1*, all of which were significantly upregulated in fibrosis Oncopig livers. Down-regulation of several miRNAs (ssc-miR-363, ssc-miR-222, and ssc-miR-106a) targeting mRNAs coding for anti-apoptotic mitochondrial genes (*BCL2L11* and *BCL2L1*) was also observed and suggests a reduction in cellular toxic insult and death corresponding to a reduction in hepatic fibrosis. Additionally, several miRNAs upregulated (ssc-miR-146b, ssc-miR-27a, ssc-miR-10b, and ssc-miR-21) and 1 downregulated (ssc-miR-222) in fibrotic Oncopig livers targeting pro-apoptotic FADD and *APAF1* genes were identified. Finally, DE miRNAs involved in pathways perturbed in human HCC were also identified (Table 1) [42].

**Table 1.** Differentially expressed miRNAs with known roles in HCC. Adapted from Xu et al. [42]

	Predicted targets	Mechanisms	logFC	p-value
miR-107	CCNE1, CDK6, CRKL, SERBP1	Proliferation, prognostic marker	−0.9045	0.000439
miR-10b	APAF1, NF1, PTEN	Migration, invasion	1.4281	0.000411
miR-1249	None	Cell growth, migration, invasion	1.3221	0.001069
miR-144	None	Proliferation, invasion, migration	1.0263	0.04285
miR-199a-3p	None	Angiogenesis, proliferation, apoptosis	0.9171	0.00025
miR-199a-5p	None	Cell growth	0.9288	0.000736
miR-199b-5p	DYRK1A, ETS1, HIF1A, MYH9, PDCD4, SET, SIRT1	EMT	1.2205	0.000205
miR-21	CAMSAP1, DDX1, MARCKSL1	No mentioned	0.8436	0.004984
miR-331-3p	None	Proliferation, apoptosis	−0.5771	0.038229
miR-340	None	Proliferation, invasion	0.8057	0.009188
miR-874	None	Proliferation, metastasis	−1.0484	0.002785

### Differential DNA methylation associated with fibrosis induction

**Figure 4.**
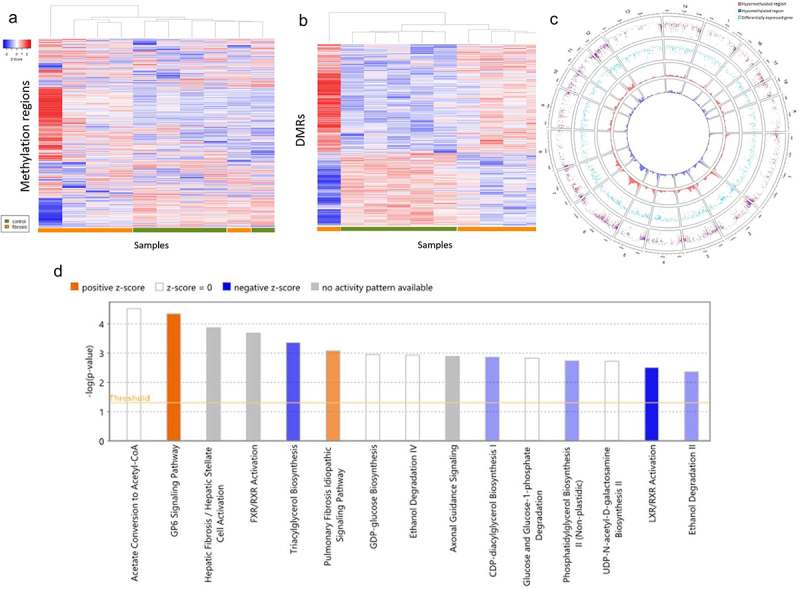
Impact of liver fibrosis induction on DNA methylation patterns. (a) Hierarchical clustering of subjects based on methylation frequency within all identified methylation regions and (b) differentially methylated regions and genes. From outermost ring to innermost ring: coordinates of differentially methylated regions within each chromosome; density of hypomethylated regions along chromosome. (d) Pathway analysis of differentially expressed genes containing at least one differentially methylated region.

In total, 7,715 differentially methylated regions (DMRs) were identified. Based on proximity, 717 DMRs were located within 10,000 bp upstream of a known gene, 3,918 DMRs were located within an annotated gene, and 175 DMRs were found to be located within 165 known 5’ untranslated regions (5’ UTRs). Hierarchical clustering based on methylation levels of all methylation regions and DMRs demonstrated partial separation of fibrosis and control arms (Figure 4(a,b)). The genomic coordinates of DMRs and DEGs were plotted for qualitative assessment of DEG-DMR overlaps to identify DM DEGs (Figure 4(c)). A total of 502 DEGs were found to contain at least one DMR (Supplementary Table S4). Pathways enriched for DM DEGs included acetate to acetyl-CoA conversion, GP6 signaling, hepatic fibrosis and stellate cell activation, and farnesoid X receptor (FXR) and retinoid X receptor (RXR) activation (Figure 4(d))

**Figure 5.**
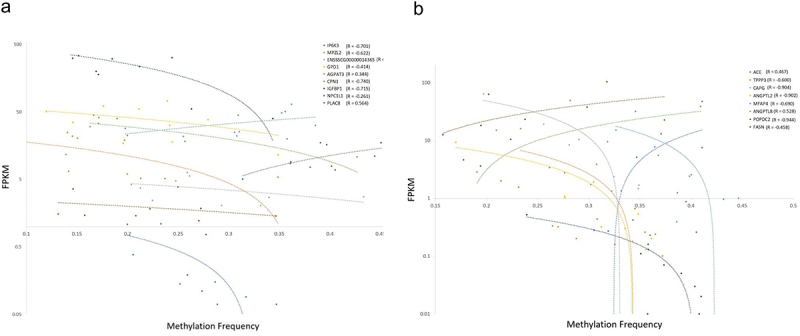
Correlation between methylation and repression in DEGs with 5; UTR DMRFs. (a) Methylation frequency versus fold-per-kilobase-million expression. (b) Methylation frequency versus fold-per-kilobase-million expression for each sample for each DEG within the top quartile of log fold-change expression.

The 175 DMR’s contained in a 5’ UTR were located within 165 unique 5’UTR’s with each 5’UTR containing between one and four DMRs. Among these 175 DMRs within 5’ UTR’s, 33 were located within the 5’ UTR of a DEG. Within this subset, methylation frequency of DMR’s and fold-per-kilobase-million expression of their respective DEG’s were compared for each subject (Supplementary Table S5). Averaged across all DMR-DEG pairs, a slight negative correlation between methylation frequency and expression was observed (*R* = −0.288), with a stronger negative correlation observed for DEGs in the highest and lowest quartile of log fold-change expression (*R* = −0.369 and −0.329, respectively; Figure 5(a,b)). Qualitatively, this set was enriched for genes involved in cholesterol and glycerolipid metabolism including pathways such as FXR/RXR activation, liver X receptor (LXR)/RXR receptor activation, and palmitate and fatty acid synthesis pathways (Supplementary Table S6).

### DM DEGs targeted by DE miRNAs

A total of seven DM DEGs that were also known targets of DE miRNAs were identified (Table 2). *ANPEP, COL1A1*, and *FOXO3* were upregulated in Oncopig fibrotic liver samples, while *EGFR, ODC1, TLR1*, and *GPD1* were downregulated in Oncopig fibrotic liver samples. *EGFR* demonstrated extensive differential epigenetic regulation, with three DMRs identified in a 51.4kbp interval within intron 2 of the gene body. Additionally, *EGFR* was also a predicted target of ssc-miR-1, which was overexpressed in fibrosis Oncopig livers. Similarly, *ODC1*, an enzyme involved in polyamine synthesis that has been implicated as an oncogene in numerous cancers [43], was also found to contain two hypomethylated regions in a 9.02kbp interval within intron 13 and was the target of an overexpressed miRNA, ssc-miR-27a. Finally, *FOXO3* was observed to have one differentially hypomethylated region within intron 2 and was associated with a under-expressed miRNA, ssc-miR-222 [44].

**Table 2.** Differentially expressed genes containing at least one differentially methylated region which were also predicted targets of a differentially expressed micro RNA. Green text indicates differential overexpression or hypermethylation, red text indicates differential underexpression or hypomethylation.

DEG (LogFC)	DEmiRNA (LogFC)	DMR (Δmethylation fraction*)
*COL1A1* (3.74)	ssc-let-7i (0.89) ssc-miR-29c (0.70)	chr12:26,393,528–26,393,590 (0.148)
*ANPEP* (1.49)	ssc-miR-1 (0.88)	chr7:55,364,806–55,365,057 (0.125)
*FOXO3* (1.08)	ssc-miR-222 (−0.69)	chr1:74,731,403–74,731,504 (−0.345)
*TLR1* (−0.79)	ssc-miR-146b (2.18)	chr8:30,173,920–30,174,107 (0.108)
*ODC1* (−1.05)	ssc-miR-27a (1.11)	chr3:126,119,837–126,120,212 (−0.136); chr3:126,128,621–126,128,864 (−0.188)
*EGFR* (−1.41)	ssc-miR-1 (0.88)	chr9:139,328,398–139,328,609 (0.172); chr9:139,374,613–139,374,778 (0.110); chr9:139,379,524–139,379,841 (−0.115)
*GPD1* (−1.42)	ssc-miR-214 (0.82)	chr5:16,008,432–16,008,495 (0.139)

*Defined as the arithmetic difference between methylation frequency in experimental and control samples.

### Multi-omics analysis identifies subset of highly discriminatory signatures

**Figure 6.**
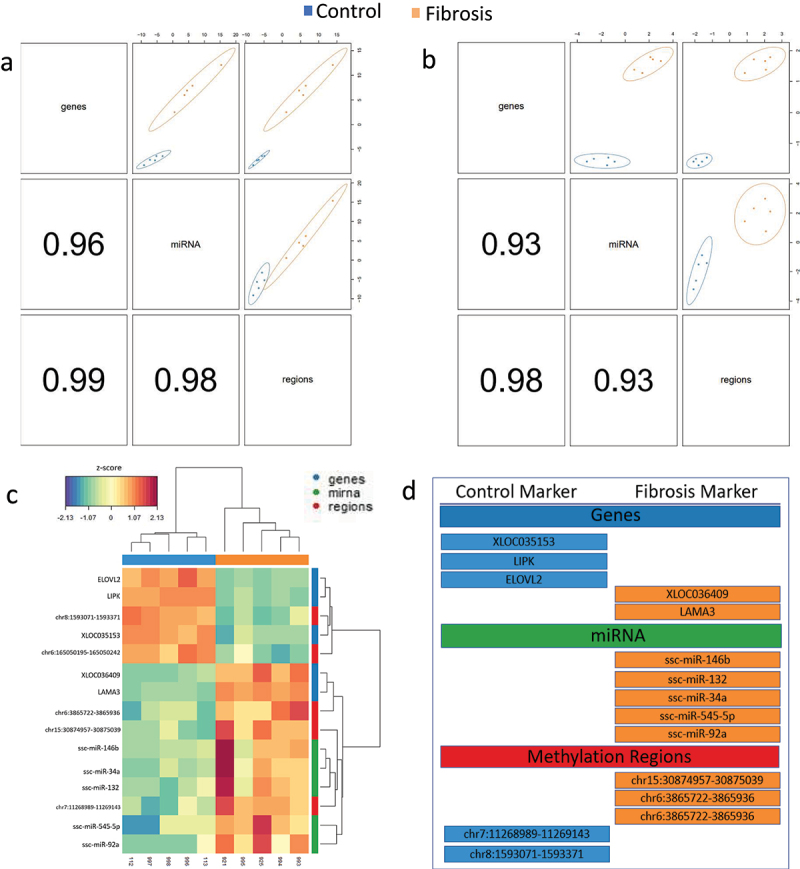
Epigenetic alterations associated with fibrosis induction identified through multi-analysis. (a) 100 variable latent space and (b) # variable latest space component analysis (above the diagonal) and correlation coefficients (below the diagonal). (C) Hierarchical clustering of subjects and variable within the five-variable latent space. (d) Control and fibrosis markers within each ‘omics data set as predicted by the five-variable model. Fibrosis markers were defined as the mRNA’s, miRNA’s, and methylation regions which were either overexpressed or hypermethylated in Oncopig fibrotic livers whereas control markers were defined as the mRNA’s, miRNA’s, and methylation regions overexpressed or hypermethylated in the control arm of the study.

Single component, supervised DIABLO latent variable analysis of mRNAs, lncRNAs, miRNAs, and methylated regions was performed to validate the single-omics analyses performed in this study and identify variables which most reliably discriminated between fibrotic and control sample. Analysis was first performed by selecting the 100 most discriminatory variables from each omics dataset to provide sufficient data for pathway analysis while maintaining sufficient separation of the control and fibrosis groups within the latent space (Figure 6(a)). All of the genes, regions, and miRNAs identified in the 100-variable analysis were also identified in their corresponding single-omics analysis. Pathway analysis of mRNAs revealed activation of hepatic fibrosis and stellate cell activation, adipogenesis, and ketogenesis pathways as well as several other liver-specific metabolic pathways. Single component, five variable analysis was then performed to identify a discrete panel of potential biomarkers associated with liver fibrosis development. A total of 5 genes, miRNAs, and methylation regions were identified which reliably discriminated the fibrosis and control groups (Figure 6(b–d)). These variables were defined as fibrosis markers if they were overexpressed or hypermethylated in Oncopig fibrotic liver samples, whereas control markers were overexpressed or hypermethylated in control samples. Identified genes included *LAMA3*, which was overexpressed in Oncopig fibrotic livers and is an essential component of the extracellular basal lamina [45]. Other gene signatures included reduced expression of *ELOVL2*, involved in long and very-long chain fatty acid elongation, and *LIPK*, a lipase involved in lipoprotein metabolism [46,47]. Finally, reduced expression of the lncRNA XLOC035153 was identified. Its partner mRNA *S1PR1*, involved in vasculogenesis and cancer cell motility via the RAC-CDC42 and ERK pathways [48], displayed reduced expression in the single-omics analysis.

## Discussion

Large animal models of liver cirrhosis and HCC are of particular interest to physicians and biomedical researchers focused on the discovery and development of novel therapeutics and procedures for the diagnosis and treatment of these diseases. The fibrosis induction approach utilized in this study results in significantly higher liver fibrosis and inflammation levels compared to healthy Oncopig liver samples based on a porcine adapted METAVIR scheme (a clinically validated tool used to diagnosis and grade human liver fibrosis/cirrhosis) [23]. Peak fibrosis severity (METAVIR F2-F3) occurs at 8 weeks post-induction followed by gradual resolution after cessation of alcohol infusion by 20 weeks post-induction. As METAVIR F4 is generally accepted to represent irreversible alcohol-induced cirrhosis clinically, further model development work is required to develop an Oncopig cirrhosis model. However, the resolution observed in this model is consistent with liver recovery that has been observed in non-cirrhotic (METAVIR F3 or lower) patients following abstinence from alcohol [49,50]. The present study therefore aimed to analyze the transcriptional changes and epigenetic regulatory mechanisms involved in hepatic fibrosis development and recovery in this porcine model of alcoholic liver disease, and to compare these changes to the known transcriptional and epigenetic alterations that drive human alcoholic liver disease, cirrhosis, and HCC.

The histopathological hallmark of cirrhosis is the widespread deposition of fibrotic bands composed of various collagen isoforms laid down by activated stellate (Ito) cells in the space of Disse within hepatic lobules. Genes encoding for alpha subunits of the laminin protein (*LAMA3, LAMA1, LAMA2*, and *LAMA4*), a heterotrimer protein which is involved in cellular adherence to extracellular matrix proteins, were overexpressed in Oncopig fibrotic livers. The laminin protein is a key protein involved in human fibrogenesis, is overexpressed in a range of neoplasms, and is positively correlated with portal pressure in compensated cirrhotic patients [51,52]. These findings, in concert with the overexpression and epigenetic regulation of *COL12A1, COL1A1, COL27A1, COL4A2, COL4A4, COL5A1*, and *COL8A2* provide evidence that the process of fibrogenesis is still ongoing at the 8-week post-induction time point at which these samples were collected. Interestingly, several isoforms of matrix metalloproteases, including *MMP2*, which acts to breakdown the collagenous extracellular matrix, were also found to be uniformly upregulated, suggesting that there is also a reparative process being carried out within the fibrotic liver [53]. As collagen isoforms, miRNAs targeting collagen isoforms, and MMP2 are all upregulated, these expression patterns collectively suggest concurrent activation of collagen synthesis and breakdown and regression pathways and is consistent with the transient fibrosis with concomitant regression previously described in this model [23]. Therefore, temporal sequencing and single-cell RNA-seq may be beneficial to better understand the timing and identify cellular subpopulations associated with both of these processes.

Metabolic derangements, namely dysfunction of sex hormone metabolism, are also a well-known hallmark of alcoholic liver disease [54]. Metabolic pathways perturbed in response to Oncopig fibrosis induction included acetate to acetyl-CoA conversion, ethanol degradation, dysregulation of bile acid, and hydroxysteroid modification. The six hydroxysteroid dehydrogenase enzymes identified as DEGs in the single-omic analysis were uniformly downregulated in Oncopig fibrotic livers. This, in addition to the overexpression of lncRNA’s partnering with enzymes involved in sex hormone metabolism, such as *HSD3B1, HSD17B4*, and *AKR1C3* suggests some level of dysregulation of these pathways. Interestingly, three of these six enzymes were isoforms of 17-β-hydroxysteroid dehydrogenase. Various isoforms of 17-β-hydroxysteroid dehydrogenase, namely *HSD17B13*, have previously been found to be associated with non-alcoholic fatty liver disease and HCC recurrence and progression [55,56].

The role of miRNA and epigenetic regulation has been studied extensively in human alcoholic liver disease and HCC. miRNAs play a direct role in the pathogenesis of alcoholic liver disease and may even serve as biomarkers for alcohol-induced inflammation [7]. In the present study, key DEGs involved in fibrosis deposition and regression displayed extensive epigenetic regulation in the form of miRNA targeting, lncRNA partnership, and differential DNA methylation. Most notable among these is *EGFR*. EGFR is a transmembrane receptor tyrosine kinase, which has been purported to play a role in human HCC development as well as liver regeneration [57]. Downregulation of *EGFR*, overexpression of a targeting miRNA, and DM of three regions within a single intron were observed in Oncopig fibrotic liver samples. Similarly, *FOXO1* and *FOXO3* were found to be under multiple layers of epigenetic control. Both of these genes were overexpressed and targeted by DE miRNAs, with *FOXO3* also being differentially methylated. These genes, which belong to the FOXO family of transcription factors, have a diverse array of functions, perhaps most notably in orchestrating the cellular stress response and promoting the homeostasis of cellular proteins through autophagy [58]. Additionally, genes associated with cellular proliferation and cell cycle regulation, such as *PTEN* and *CDK6*, were among the genes most frequently targeted by DE miRNAs. While it is difficult to definitively characterize from ‘omics data collected from a single time point, taken together, these findings may suggest that the pathways that constitute the repair process following alcohol-induced liver injury may be initiated and terminated prior to initiation of cellular proliferation and regeneration. This ‘staggering’ of these pathways may be accomplished, at least in part, by a host of epigenetic control mechanisms. For example, *FOXO* overexpression and relative *EGFR, PTEN*, and *CDK* downregulation in the acute phase of alcoholic liver injury may serve to allow time for cellular or nuclear repair before advancement through the cell cycle and subsequent cellular proliferation.

Multi- and single-omics analysis performed in this study was largely concordant. For instance, pathway analysis of both single- and multi-omics DEGs demonstrated broad involvement of hepatic fibrosis, hepatic metabolic, and proinflammatory and anti-inflammatory signaling pathways. DE miRNAs were also consistent across both types of analyses, implicating suppression of several collagen isoforms, differential regulation of pro- and anti-apoptotic genes such as *BCL2* and *FADD*, and cell cycle regulating and anti-proliferative genes such as *CDK6* and *PTEN*. Five variable multi-omics analysis provided more granular understanding of the core differences in expression between groups, with *ELOVL2, LIPK, LAMA3*, and lncRNAs partnered with *UHRF1BP1L* and *S1PR1* found to most reliably discriminate the control and fibrosis groups. This 5-variable analysis provides a small list of potential multi-omics biomarkers that could be used to assess disease severity, although additional follow-up studies are required to confirm this.

Both *ELOVL2* and *LIPK*, which were identified via both single and multi-omic analyses and displayed reduced expression in Oncopig fibrotic liver samples, are involved in lipid and lipoprotein metabolism, respectively. More specifically, *ELOVL2* which displayed the largest reduction in expression between groups, is a very long-chain fatty acid elongase. Its related isoforms, namely *ELOVL6*, have been found to have a bi-directional relationship with the pathogenesis of non-alcoholic steatohepatitis in humans and mice [59,60]. *LIPK* codes for an acid lipase, lipase family member k, which has not previously been associated with alcoholic liver disease, but has been described as being expressed in human epidermal tissues and plays a role in the barrier function of differentiated keratinocytes [61]. These findings, in concert with the DE and differential 5’UTR methylation of genes involved in lipid homeostasis identified in this study, such as *FASN, LCAT*, and *APOB*, suggest an alcohol-induced disruption of lipid metabolism in Oncopig fibrotic livers, as is seen in human alcoholic liver disease.

Finally, the partner gene of lncRNA identified via single and multi-omic analyses, *S1PR1*, is of particular interest in this study. It has not been previously described as involved in cirrhosis induction or HCC progression, but is known to play a broad role in oncogenesis across multiple cancer types. *S1PR1* was found to be overexpressed and targeted by a DE miRNA, ssc-miR-106a. *S1PR1* promotes both neovascularization and decreased cell-to-cell adhesion, two of the pathological hallmarks necessary for oncogenesis and metastasis, although this gene has never been directly implicated as an oncogene for HCC or a cirrhosis associated mutation [48]. Additionally, given that the partner lncRNA to *S1PR1* was down-regulated in Oncopig fibrotic liver samples, and that the gross and microscopic changes observed in prior studies showed evidence of fibrosis regression without evidence of frank cirrhotic change, it is reasonable to surmise that the hepatic insult delivered by ethanol infusion was not sufficient to induce an *S1PR1* mediated irreversible or neoplastic process.

Limitations of this study include the observed liver recovery in this model, indicating further works is required to develop protocols aimed at inducing METAVIR F4 liver cirrhosis in pigs, for example through exposure to chronic alcohol feeding in combination with intrahepatic ethanol injection. In addition to the need for continued model development, incorporation of more comprehensive cross-species comparisons would provide valuable insights into the translational relevance of porcine and other preclinical animal models of liver cirrhosis. Other limitations include an all-female cohort, lack of sequencing data from different time points during the fibrosis induction process, and the relatively small sample size. Follow-up analyses of samples taken at 1- or 2-week intervals pre- and post-fibrosis induction could be helpful to elucidate the temporal evolution and interrelationships of the pathways identified in this study. The ability to evaluate differential methylation in the context of regulatory features including promoters, enhancers, and silencers was also limited by the lack of functional annotation available for the pig genome.

Finally, as cirrhotic liver patients have a high risk of developing HCC, additional long-term follow-up studies are required to determine the rate and frequency at which HCC tumor development is observed in pigs with alcohol-induced liver fibrosis. Regardless, as the Oncopig is capable of forming HCC and other tumor types in a controlled and timely fashion, this work sets the stage for future studies in which HCC tumors can be induced in fibrotic Oncopig livers to more closely mimic human disease. Therefore, while these limitations do hinder the generalizability of this study, the findings of the present study demonstrate consistency between Oncopig and human transcriptional patterns and epigenetic regulation in the context of alcoholic liver disease, highlighting the translational relevance of this porcine alcohol-induced liver fibrosis model.

## Supplementary Material

Supplement2.xlsx

Supplement3.xlsx

Supplement5.xlsx

Supplement4.xlsx

Supplement6.xlsx

Supplement1.xlsx

## Data Availability

The data that support the findings of this study are available in the NIH Sequence Read Archive (SRA) at the following URL: ID 629,513 - BioProject – NCBI (nih.gov)
